# Complete Genome Sequence of a Chicken Embryo Fibroblast-Adapted Attenuated Infectious Bursal Disease Virus Isolate from India

**DOI:** 10.1128/genomeA.00352-16

**Published:** 2016-05-12

**Authors:** T. M. A. Senthilkumar, C. V. Priyadharsini, P. Raja, K. Kumanan

**Affiliations:** a Department of Animal Biotechnology, Madras Veterinary College, TANUVAS, Chennai, India; b Faculty of Basic Sciences, Madras Veterinary College, TANUVAS, Chennai, India

## Abstract

Infectious bursal disease virus is an avian pathogen that causes huge morbidity and mortality in the poultry sector all over the world. Here, we report the full-length genome sequence of an Indian strain, MB11/ABT/MVC/2016, isolated from a commercial broiler flock. This is a first report of a complete genome sequence of infectious bursal disease virus from India.

## GENOME ANNOUNCEMENT

Infectious bursal disease virus (IBDV) is a nonenveloped virus, with a single capsid shell of icosahedral symmetry composed of 32 capsomers with diameters of 55 to 60 nm. The virus genome consists of two segments (A and B) of double-stranded RNA. IBDV is a member of the family *Birnaviridae*, genus *Avibirnavirus* (1). It remains one of the most important contributors to the huge economic loss to poultry farmers worldwide due to its immunosuppressive nature even after utilizing the proper vaccination schedule (2). All pathogenic IBDV (serotype 1) strains can be divided into classic, variant, and very virulent strains, according to their antigenic and pathogenic nature (3). However, different antigenic variants of IBDV have been reported, and they are responsible for the subclinical form of the disease and lead to various degrees of immunosuppression. Here, we report for the first time the full-genome sequence of an IBDV isolate from a clinical outbreak in a commercial broiler flock.

The IBDV isolate was obtained during 2011 from a 4-week-old commercial broiler flock suffering from respiratory problems with high mortality. Total RNA was isolated from the bursal tissue using TRIzol reagent (RNAiso Plus; TaKaRa). The extracted RNA was subjected to synthesis of cDNA using a commercial cDNA synthesis kit (RevertAid H Minus first-strand cDNA synthesis kit; Thermo Scientific, USA). The presence of IBDV was diagnosed by agar gel immunodiffusion (AGID) using specific hyperimmune serum (Charles River Avian Vaccine Services) and reverse transcriptase PCR (RT-PCR) by amplifying the VP2 hypervariable region (4). Further, this strain was continuously passaged (58 passages) in chicken embryo fibroblast primary culture, complete genome sequencing was carried out by RT-PCR using overlapping consensus primers, and the PCR-amplified products were gel purified for direct sequencing in both directions by M/s Shrimpex Biotech, Chennai, India. Sequences were compiled and edited using the SeqMan program (Lasergene). Multiple sequence alignments were performed with MEGA5 (5), and a phylogenetic tree was constructed using PhyML.

The size of the IBDV full genome reported here is 3,260 nucleotides (nt) in segment A and 2,827 nt in segment B. The coding region of segment A contains two open reading frames that encode VP5 (145 amino acids [aa]) and a VP2-VP4-VP3 polyprotein (1,012 aa); segment B encodes VP1 (879 aa), the viral RNA dependent RNA polymerase.

Phylogenetic analysis of the VP2 hypervariable region indicates that the virus belongs to the attenuated IBDV strain lineage. This was confirmed by the presence of unique and conserved amino acid changes at Q253H, D279N, A284T, and S330A. The observation of alterations of amino acids at positions 253 and 284 occurred in both tissue culture adaptation and virus attenuation in specific-pathogen-free (SPF) chickens (6).

Comparative analysis of the complete genome segment A shows higher nucleotide similarity (99.4%) with attenuated strain Gt (accession no. DQ403248). In the case of segment B, the highest similarity (99.9%) was found with attenuated strain Gt (accession no. DQ403249). The complete genome sequences of many such IBDV isolates from India will help better understand virus epidemiology and contribute to the effective control of IBDV infection.

### Nucleotide sequence accession numbers.

The full-length sequence of the isolate MB11/ABT/MVC/2016 has been deposited in GenBank under the accession numbers KU891986 (segment A) and KU891987 (segment B).
